# Associations of folic acid supplements and dietary folate intake with gestational diabetes mellitus: complementary evidence from a multimethod investigation

**DOI:** 10.3389/fnut.2026.1665917

**Published:** 2026-02-16

**Authors:** Yanyan Hu, Yifei Wang, Cheng Xue, Yifang Hu, Dan Wang, Jizheng Wang, Yanfei Mo, Wensong Zhang, Ting Ge, Wenjie Ma, Ying Lu, Yun Liu, Shan Lu

**Affiliations:** 1 Department of Geriatrics, The First Affiliated Hospital of Nanjing Medical University, Nanjing, Jiangsu, China; 2 Division of Geriatric, Liyang People’s Hospital, Liyang Branch Hospital of Jiangsu Province Hospital, Changzhou, China; 3 Department of Clinical Medical Research, The Friendship Hospital of Ili Kazakh Autonomous Prefecture, Yining, Xinjiang, China; 4 Nanjing Pukou District Chinese Medicine Hospital, Nanjing, Jiangsu, China; 5 Department of the Core Facility, The First Affiliated Hospital of Nanjing Medical University, Nanjing, Jiangsu, China; 6 Women and Children Department of the First Affiliated Hospital of Nanjing Medical University, Nanjing, Jiangsu, China

**Keywords:** cross-sectional study, folate, folic acid, gestational diabetes mellitus, NHANES

## Abstract

**Objective:**

The relationships between folic acid supplementation, folate intake, and GDM remain controversial. We conducted a preliminary investigation using a multimethod approach integrating a retrospective cohort study, Mendelian randomization, and dose–response analysis to explore this association.

**Methods:**

We examined the relationship between folic acid supplement use (including a combination preparation) and the GDM risk in a retrospective cohort of 10,479 pregnant women receiving care at Jiangsu Provincial People’s Hospital using multivariable logistic regression analysis. MR analysis provides genetic support for a potential causal link between genetically predicted folic acid supplement use and GDM. A cross-sectional analysis of 3,680 pregnant women in the National Health and Nutrition Examination Survey (NHANES) evaluated total folate intake and dietary folate equivalents (DFEs) via 24-h dietary recall; multivariable logistic regression and restricted cubic spline models were used to characterize associations and generate dose–response curves. The models were adjusted for age, BMI, race or ethnic origin, education, and smoking history. Subgroup analyses were performed to assess potential effect modifications.

**Results:**

In this retrospective cohort study, compared with non-users, folic acid supplement users had a 46.2% greater likelihood of having GDM (OR = 1.46, 95% CI: 1.339–1.595; *p* < 0.001). MR analysis supported a potential causal association between genetically predicted folic acid products and GDM risk (OR = 1.40, 95% CI 1.17–1.67, *p* < 0.001). In the NHANES cohort, higher total folate (OR = 1.42, 95% CI: 1.05–1.92, *p* = 0.02) and DFE intake (OR = 1.61, 95% CI: 1.23–2.10, *p* < 0.001) were linked to an increased GDM risk, with non-linear dose–response inflection points at approximately 445 μg/day and 582 μg/day, respectively. These associations were generally maintained after multivariable adjustment, and subgroup analyses revealed consistent trends toward an increased risk.

**Conclusion:**

This multimethod study indicates that both supplemental folic acid and dietary folate intake may be associated with an increased GDM risk. These observations support the need for additional research to better understand the potential impact of current recommendations on prenatal folate levels.

## 1 Introduction

Gestational diabetes mellitus (GDM) is a metabolic disorder characterized by glucose intolerance that first occurs during pregnancy. Its prevalence has been increasing globally, making it a significant public health concern that affects maternal and fetal health (1). GDM not only increases the risk of adverse pregnancy outcomes such as macrosomia, preterm birth, and cesarean delivery but also predisposes mothers to long-term complications, including type 2 diabetes and cardiovascular diseases, thereby imposing a substantial economic burden on healthcare systems (2). Folate, a key nutrient for preventing fetal neural tube defects, is widely recommended for supplementation during pregnancy (3–5). However, emerging studies suggest that folic acid supplementation may have potential adverse effects on glucose metabolism during pregnancy, possibly increasing the risk of GDM, leading to ongoing debates regarding prenatal nutritional interventions (6, 7).

The existing evidence on the relationship between folic acid and folate intake and the GDM risk remains contradictory. Some studies have reported that higher folate intake is associated with improved glucose metabolism and a reduced risk of GDM (8, 9). Conversely, other studies have linked high-dose folic acid supplementation to an increased risk of GDM (10–13). These findings suggest that appropriate folate intake may increase insulin sensitivity and regulate glucose metabolism, potentially exerting protective effects. However, the dose–response relationship of folate may follow a threshold effect. The inconsistencies among studies may stem from differences in the study design, sample selection, measurement, and adjustment for confounding factors, leading to a lack of consensus on the optimal intake threshold and the precise mechanisms underlying the effects of folate on the GDM risk. Consequently, a considerable research gap and controversy still exist regarding the non-linear association and potential causal relationship between folic acid and folate intake and the GDM risk, necessitating more rigorous and diversified research approaches.

In efforts to investigate the relationship between folic acid and folate intake exposure and gestational diabetes mellitus (GDM), the distinct forms of vitamin B9 involved must be clarified. Folate refers to naturally occurring vitamin B9 found in foods such as leafy vegetables, legumes, nuts, fruits, animal liver, and whole grains, whereas folic acid denotes the synthetic form used in dietary supplements and food fortification, which is more bioavailable than natural folate (14). Accurately distinguishing between these two forms helps clarify exposure sources in studies examining their associations with GDM (15). In addition, total folate intake represents the combined amount obtained from naturally occurring dietary sources and synthetic folic acid obtained from fortified foods and/or supplements. Dietary folate refers exclusively to the form naturally present in foods such as dark green vegetables, legumes, and fruits. Dietary folate equivalents (DFEs) serve as a standardized unit that accounts for the higher bioavailability of synthetic folic acid than natural folate and adjusts for these differences, thereby providing a more accurate reflection of the physiological activity of folate in the body.

Our study employed a multifaceted approach integrating evidence from three complementary methodologies across diverse populations to comprehensively investigate this relationship. Rather than aiming for direct cross-population comparisons, this study seeks to triangulate evidence from observational, genetic, and dose–response perspectives to determine whether a consistent signal for a folate–GDM association emerges across different methodological approaches. First, we analyzed clinical data from a retrospective cohort of 10,479 pregnant women to examine the association between folic acid supplement use and the GDM risk. Second, we conducted Mendelian randomization analyses using genetic variants associated with genetically predicted folic acid products to assess potential causality. Third, we utilized data from the NHANES cohort with detailed 24-h dietary recalls to quantify the dose–response relationships between folate intake (including total folate intake, dietary folate intake, and DFEs) and the GDM risk. This tripartite design enables the triangulation of evidence from clinical, genetic, and nutritional epidemiological perspectives, providing a comprehensive assessment of the folate–GDM relationship while compensating for the inherent limitations of each methodological approach. We acknowledge that retrospective and MR analyses provide preliminary evidence, and these findings warrant further validation through rigorously designed randomized controlled trials (RCTs) to establish causality. We must emphasize that this multimethod, triangulation analysis is fundamentally exploratory and hypothesis-generating. Its primary aim is to illuminate potential associations and biological plausibility, thereby laying the groundwork for future definitive studies; it is not intended to serve as a basis for clinical guidance at this stage.

## 2 Materials and methods

### 2.1 Research design and data source

Three distinct data sources were leveraged to comprehensively investigate the link between folate use and GDM. The primary source was a retrospective cohort of pregnant individuals from Jiangsu Provincial People’s Hospital, China, from which we obtained real-world clinical records on folic acid supplement use (e.g., whether folic acid supplements were used, the number of bottles, and the duration of use) and GDM outcomes. Due to the lack of precise assessment and documentation of folic acid levels in the database, a dose–response analysis could not be conducted. We incorporated an MR analysis using folic acid product-related GWAS summary data from European populations to complement these findings and assess potential causality while minimizing confounding. This genetic approach provides a preliminary line of evidence on the genetically predicted folic acid–GDM relationship. We included the NHANES dataset, which provides detailed dietary assessments of folate intake, including total folate intake, dietary folate intake, and dietary folate equivalents (DFEs), to address this limitation. These high-resolution data allow a more precise evaluation of dose–response and non-linear associations, complementing the binary exposure measurement in the retrospective cohort. Together, these sources were selected not for comparative purposes but to evaluate whether a consistent association emerges across observational, genetic, and dose–response perspectives despite differences in methodology and population backgrounds. Importantly, folic acid supplementation refers to the intake of synthetic folic acid from supplements, whereas dietary folate intake refers to folate obtained from natural food sources. Total folate intake represents the sum of supplemental and dietary folate, and dietary folate equivalents (DFEs) are used as the standardized unit for quantifying and reporting all intake values for total folate intake and dietary folate intake.

### 2.2 Retrospective cohort analysis

We conducted a retrospective cohort study using clinical data from pregnant individuals who visited obstetric outpatient clinics or who were admitted to the obstetric ward at Jiangsu Provincial People’s Hospital, China, between 2020 and 2024. The cohort comprised 10,479 participants with documented folic acid supplementation information (including the use status, number of bottles, and duration) and clinical measurements (height, weight, BMI, and GDM diagnosis). The variable “folic acid use” was defined as a binary measure (yes/no) indicating whether a pregnant woman received and consumed folic acid tablets provided by local maternal and child healthcare institutions at the time of establishing her perinatal health record. The “number of bottles” was used as an administrative unit to quantify folic acid exposure in this system. Each bottle contained 31 tablets of folic acid, corresponding to approximately 1 month of supplementation. In this cohort, folic acid exposure primarily reflects government-issued single folic acid tablets. A minority of participants reported using multivitamins containing folic acid; however, this group represents only a small fraction and is not the main exposure. The inclusion criteria were (1) delivery at Jiangsu Provincial People’s Hospital during the study period, (2) a confirmed diagnosis of gestational diabetes mellitus (GDM) or non-GDM status based on clinical records, and (3) availability of complete antenatal records, including details on folic acid supplementation. The exclusion criteria included the following: (1) preexisting diabetes mellitus diagnosed before pregnancy, (2) incomplete or missing folic acid supplementation records, and (3) the absence of critical clinical variables (GDM outcome). We compared patterns of folate use between women with and without GDM. The prespecified subgroup analysis stratified participants by trimester of exposure (early stage: 1–12 weeks; midterm stage: 13–24 weeks; later stage: 25–42 weeks) to assess effect modifications. Multivariable logistic regression models were applied to control for potential confounders. Model 1 was adjusted for age, height, and weight. Model 2 was further adjusted for education level and the trimester of exposure. Group characteristics are summarized as the means ± SDs or frequencies (%) and were compared using t tests or chi-square tests. Univariable logistic regression was used to estimate crude ORs and 95% CIs. Additionally, this retrospective cohort study adhered to the STROBE statement—the checklist of items that should be included in reports of cohort studies (Supplementary Table S6).

### 2.3 Mendelian randomization analysis

We performed a two-sample Mendelian randomization (MR) analysis using publicly available genome-wide association study (GWAS) summary statistics to further validate the observational associations detected in the retrospective cohort of Chinese women.

#### 2.3.1 Data sources

Exposure Data: Genetic associations with folic acid supplementation were obtained from the UK Biobank (GWAS ID: ukb-b-288), comprising 462,900 individuals (3,883 patients and 459,050 controls). Outcome Data: Genetic associations with gestational diabetes mellitus (GDM) were sourced from the FinnGen consortium (GWAS ID: finn-b-GEST_DIABETE), which included 123,600 participants (5,687 GDM patients and 117,892 controls).

#### 2.3.2 Selection of genetic instruments

The selection of instrumental variables (IVs) was based on the three core assumptions of MR. Single-nucleotide polymorphisms (SNPs) were selected according to the following rigorous criteria to satisfy the first assumption that the genetic instruments must be robustly associated with the exposure: (1) single-nucleotide polymorphisms (SNPs) were significantly associated with folate supplementation (*p* < 5 × 10^−8^); (2) independent SNPs were selected using linkage disequilibrium (LD) pruning (*r*^2^ < 0.001); and (3) SNPs with an F-statistic >10 were retained to ensure strong instrument validity. The exposure in the MR analysis corresponded to genetically predicted folic acid supplement use derived from GWAS data. This approach reflects the propensity to take folic acid products rather than circulating folate concentrations or metabolic status. Accordingly, MR estimates should be interpreted in the context of supplement-taking behavior and may be influenced by behavioral or socioeconomic factors.

#### 2.3.3 MR analysis methods

The primary causal effect was estimated using the inverse-variance weighted (IVW) method and a random-effects model. The analysis was supplemented with four additional MR methods to ensure the robustness of the findings: MR–Egger regression, the weighted median estimator, the simple mode, and the weighted mode. The consistency of the causal signal was evaluated based on the concordance in the direction of effect estimates (β) and statistical significance (*p* < 0.05) across these methods.

#### 2.3.4 Sensitivity analyses

Comprehensive sensitivity analyses were performed to validate the assumptions. Horizontal pleiotropy was assessed using the MR–Egger intercept test and the global test of the MR–Pleiotropy RESidual Sum and Outlier (MR-PRESSO) method; the absence of significant pleiotropy was indicated by a non-significant intercept (*p* > 0.05) and a non-significant global test result (*p* > 0.05), respectively. Heterogeneity among the SNP-specific estimates was quantified using Cochran’s Q statistic. A leave-one-out analysis was conducted by iteratively removing each SNP and recalculating the IVW estimate to ensure that the overall results were not driven by any single influential SNP. The instrumental variables (single nucleotide polymorphisms, SNPs) used in this study were derived from the aggregated data of published genome-wide association studies (GWASs). These aggregated data have undergone strict quality control before being made public, and no values were missing at the individual level. All Mendelian randomization analyses were completed using R software (version 4.3.1), TwoSampleMR, and the Mendelian randomization package.

#### 2.3.5 Reporting and quality control

Effect estimates are reported as odds ratios (ORs) with 95% confidence intervals (CIs). This MR analysis was planned and reported according to the STROBE-MR guidelines to ensure methodological rigor and transparency (Supplementary Table S7). In the MR analysis, the exposure phenotype (“folic acid product”, UKB-B-288) is derived from a binary self-reported supplement use variable, but the corresponding GWAS in the OpenGWAS platform is modeled using a liability threshold framework. As indicated in the metadata (“unit = SD”), the SNP–exposure associations are standardized and represent the effect per one standard deviation (SD) increase in the genetically predicted liability of folic acid supplementation rather than a binary transition from non-use to use. This standardization is typically applied for binary exposures and ensures that the exposure is analyzed as a continuous latent trait, which is consistent with MR assumptions. Given that folic acid supplementation is a behavioral exposure, we acknowledge that MR analyses of behavioral traits are inherently more susceptible to measurement error and potential horizontal pleiotropy. This contextual consideration has been incorporated into our interpretation of the MR findings.

### 2.4 Cross-sectional validation analysis using NHANES data

We performed a cross-sectional analysis of NHANES data from the 2007–2018 cycles to further validate the associations in the retrospective cohort of Chinese women. The NHANES uses a stratified, multistage probability sampling design to obtain a nationally representative sample of the nutritional and metabolic status of the non-institutionalized U. S. adult population. All the protocols were approved by the National Center for Health Statistics Ethics Review Board, and the participants provided written informed consent. All analyses incorporated the dietary day one sample weight (WTDRD1), which is specifically designed to produce nationally representative estimates from the first 24-h dietary recall interview. All analyses incorporated the appropriate sampling weights, stratification variables (SDMVSTRA), and primary sampling units (SDMVPSU) to account for the complex survey design of the NHANES. These analyses were performed using the survey package in R, which is specifically designed to produce unbiased national estimates and correct standard errors for such designs, thereby ensuring the statistical validity of the findings. In the NHANES dataset, folate exposure was assessed through 24-h dietary recall interviews, which provide detailed quantitative measures of folate intake from all dietary sources. For the present analysis, we utilized only the first 24-h dietary recall for each participant to maintain data consistency and align with the standard epidemiological practices commonly employed in NHANES-based research. This approach minimizes potential within-person correlation and ensures comparability with previous studies. We extracted three specific folate variables: total folate intake (representing the sum of intake of naturally occurring dietary folate and synthetic folic acid from fortified foods), dietary folate (referring exclusively to the intake of naturally occurring folate from food sources), and DFEs, which adjust for the differential bioavailability between natural folate and synthetic folic acid using the standard conversion. The GDM status was classified using the questionnaire item RHQ162. A limitation of this approach is that the GDM status was based on self-reports (RHQ162), which may be subject to recall bias, especially among respondents surveyed several years after their pregnancy. Key covariates, including age, BMI, race or ethnic origin, education level, and smoking status, were obtained from the demographic, dietary, and examination modules. We applied 1:3 propensity score matching (PSM) between women with and without GDM using nearest-neighbor matching without replacement to control for confounding. Propensity scores were estimated via logistic regression incorporating the covariates described above. The matched cohort was then used for subsequent multivariable logistic regression and restricted cubic spline analyses to characterize dose–response patterns of folate intake and the GDM risk, with subgroup analyses planned to explore effect modifications.

### 2.5 Study population

The inclusion criteria were as follows: (1) participants from six NHANES cycles (2007–2018) spanning 12 years; (2) clinical diagnosis of GDM, determined via the questionnaire item RHQ162: “During pregnancy, were you ever told by a doctor that you had diabetes?” (3) complete data on folate levels and relevant covariates. Adults aged ≥20 years were included in the analysis. The exclusion criteria included the following: (1) non-pregnant individuals or those lacking GDM diagnostic information, and (2) missing data on folate-related indicators (total folate intake, dietary folate intake, and DFEs) or key covariates.

### 2.6 Assessment of folate levels

Dietary folate intake data were extracted from the NHANES database to evaluate their relationship with health outcomes. Folate intake was assessed using a 24-h dietary recall method and calculated using the U. S. Department of Agriculture (USDA) dietary database. The DFE approach was used to adjust for the bioavailability of folate from different sources, ensuring sample representativeness and measurement standardization (16–18). Continuous variables, including dietary folate intake, the dietary folate equivalents (DFE), and total folate intake of participants, were categorized into quartiles (Q1–Q4) based on the distribution within the current study population. The cut-off points were defined at the 25th, 50th, and 75th percentiles, corresponding to Q1 (lowest), Q2, Q3, and Q4 (highest). This approach is a standard practice in nutritional epidemiology to explore relationships between nutrient intake and health outcomes and has been commonly employed in studies utilizing NHANES data (19, 20). Folate exposure was categorized into quartiles as follows: total folate intake—Q1 (<206 mcg), Q2 (206–307 mcg), Q3 (307–435 mcg), and Q4 (>435 mcg); dietary folate intake—Q1 (<109 mcg), Q2 (109–165 mcg), Q3 (165–240 mcg), and Q4 (>240 mcg); and folate as DFEs—Q1 (<257 mcg), Q2 (257–397 mcg), Q3 (397–563 mcg), Q4 (>564 mcg). The quartile distributions of the three folate variables validate their theoretical relationships (9, 21, 22). Dietary folate was established as the baseline (Q4: >240 mcg), total folate was substantially higher (Q4: >435 mcg), and DFEs exceeded both (Q4: >564 mcg) because of bioavailability weighting. This coherent progression supports the biological plausibility of the observed folate–GDM associations.

### 2.7 Definition of gestational diabetes mellitus

The GDM diagnosis was obtained from the NHANES 2007–2018 cycles using the health questionnaire item RHQ162: “During pregnancy, were you ever told by a doctor or other health professional that you had diabetes, sugar problems, or gestational diabetes? Please do not include diabetes diagnosed before pregnancy.” Additionally, participants were asked, “How old were you when you were first told you had diabetes during pregnancy?” Women who answered “yes” were classified as having a history of GDM, while those who answered “no” were categorized as non-GDM. Participants who responded “uncertain,” “refused,” or “did not know” were excluded from the analysis (23).

### 2.8 Covariates

The models were adjusted for multiple confounding factors to ensure the stability of the linear relationship. The demographic variables included age, race or ethnic origin (non-Hispanic white/Non-Hispanic black/Other Race/Ethnicity), and education level (less than high school/high school graduate/college or higher). Lifestyle factors included smoking status (never/former/current) and alcohol consumption (never/former/light/moderate/heavy) (24). The metabolic indicators included hypertension (yes/no), hyperlipidemia (yes/no), and body mass index (BMI). BMI was categorized according to the World Health Organization (WHO) standard as follows: underweight (<18.5 kg/m^2^), normal weight (18.5–24.9 kg/m^2^), overweight (25.0–29.9 kg/m^2^), obesity class I (30.0–34.9 kg/m^2^), obesity class II (35.0–39.9 kg/m^2^), and obesity class III (≥40.0 kg/m^2^) (25–28). Nutritional cofactors included vitamin B12 levels, additional vitamin B12 supplementation, and energy, iron, zinc, and selenium levels (29).

### 2.9 Statistical analysis

Categorical variables are reported as frequencies (%), whereas continuous variables are presented as the means ± standard deviations (SDs) or medians (interquartile ranges, IQRs). The association between folate intake and GDM was first assessed using an unadjusted logistic regression model (crude model). Four multivariable logistic regression models were subsequently constructed with increasing levels of adjustment. Model 1 was adjusted for total folate intake, alcohol consumption, smoking, and glycated hemoglobin (HbA1c) levels. Model 2 was further adjusted for race or ethnic origin and education level. Model 3 was further adjusted for BMI category, hyperlipidemia, hypertension, and vitamin B12 levels. Model 4 was fully adjusted for additional vitamin B12 supplementation and energy, iron, zinc, and selenium levels. Subgroup analyses were conducted to explore potential heterogeneity in the folate–GDM association across different population strata. Restricted cubic splines (RCSs) were applied to model the potential non-linear association between folate intake and the log-odds of GDM. An RCS model was used to fit the dose–response relationship between folate intake and GDM (30, 31). Continuous variables were Winsorized at the 1st and 99th percentiles to ensure robust estimates from the model and mitigate the potential influence of extreme values while minimizing information loss. The significance of the non-linear relationship was formally tested using a likelihood ratio test, which compared the model with the RCS terms against a simpler model containing only a linear term for folate intake. Participants with complete data for all the variables of interest were included in the final analysis (complete-case analysis). Missing data were handled using a complete-case analysis. This approach was justified by the low proportion of missing data for the primary exposure and outcome variables (less than 5%) and the absence of evidence indicating that missingness was non-random or associated with key study variables. Therefore, data imputation was not performed, as it was unlikely to influence the substantive conclusions. All the statistical analyses were conducted using R version 4.3.1 with the survey, rms, and nhanesR packages. A two-sided *p*-value of <0.05 was considered to indicate statistical significance. The NHANES analysis was planned and reported according to the STROBE Statement—Checklist of cross-sectional studies to ensure methodological rigor and transparency (Supplementary Table S8).

### 2.10 Ethics statement

This study was approved by the Ethics Committee of the First Affiliated Hospital of Nanjing Medical University (Jiangsu Province Hospital) (Ethical Approval No. 2024-SR-500). The research, titled “Precision Management of Gestational Diabetes Mellitus Based on Big Data: A Cohort Study”, was conducted in accordance with the ethical principles outlined in the Declaration of Helsinki. The current retrospective cohort analysis was explicitly included in the original ethics-approved protocol. All participants provided informed consent prior to enrolment. The secondary analysis of the GWAS sequencing data and NHANES database utilized publicly available, deidentified datasets, which were exempt from additional ethical review per institutional guidelines. Data usage adhered to the original studies’ ethical protocols and access policies.

## 3 Results

### 3.1 Baseline characteristics of the retrospective cohort of Chinese women

In the retrospective cohort analysis of women treated at Jiangsu Provincial People’s Hospital, we included 10,479 pregnant individuals, comprising 5,421 women with gestational diabetes mellitus (GDM) and 5,058 without-GDM controls. The groups were well balanced in terms of sample size, with a nearly 1:1 ratio (GDM 51.7% vs. without-GDM 48.3%), minimizing potential bias from an unequal group distribution. Compared with without-GDM controls, women with GDM had higher prepregnancy weights (*p* < 0.001), while height differed significantly between the groups (*p* = 0.026) (Table 1). Other baseline characteristics are detailed in Table 1.

**Table 1:** Baseline characteristics of participants in the hospital-based retrospective cohort study.

Characteristics	Total (*N* = 10,479)	Without GDM (*N* = 5,058)	GDM (*N* = 5,421)	*P*-value
Age, mean ± sd	29.8 ± 5.1	28.4 ± 4.8	31.2 ± 5.1	<0.001
Height, mean ± sd	161.80 ± 5.11	161.69 ± 5.131	161.92 ± 5.0926	0.866
Weight, mean ± sd	59.07 ± 9.75	58.109 ± 9.279	59.967 ± 10.11	<0.001
BMI classification (kg/m^2^), *n* (%)				<0.001
Underweight (<18.5 kg/m^2^)	622 (5.9%)	347 (3.3%)	275 (2.6%)	
Normal (18.5–24.9 kg/m^2^)	6,980 (66.6%)	3,503 (33.4%)	3,477 (33.2%)	
Overweight (25.0–29.9 kg/m^2^)	486 (4.6%)	177 (1.7%)	309 (2.9%)^*^	
Obese Class I (30.0–34.9 kg/m^2^)	1,598 (15.3%)	709 (6.8%)	889 (8.5%)^*^	
Obese Class II (35.0–39.9 kg/m^2^)	606 (5.8%)	258 (2.5%)	348 (3.3%)^*^	
Obese Class III (≥40.0 kg/m^2^)	187 (1.8%)	64 (0.6%)	123 (1.2%)^*^	
Education, *n* (%)				0.085
Below junior high school	1,875 (35.7%)	864 (17.1%)	1,011 (18.6%)	
High school graduation	1,406 (26.8%)	658 (13.0%)	748 (13.8%)	
College or above	1,746 (33.4%)	892 (17.6%)	854 (15.8%)	
Whether to take folic acid, *n* (%)				<0.001
No	2,742 (26.1%)	1,407 (13.4%)	1,335 (12.7%)^*^	
Yes	7,737 (73.8%)	3,651 (34.8%)	4,086 (39%)^*^	
Days of use, median (IQR)	90 (60, 146.8)	90 (60, 150)	90 (60, 143.5)	0.078
Dosage (bottle), median (IQR)	2 (2, 4)	2 (2, 4)	2 (2, 4)	0.079
Trimester (week), *n* (%)				<0.001
1–12 week	4,965 (77.3%)	2,599 (40.5%)	2,366 (36.8%)^*^	
13–24 week	1,415 (22%)	696 (10.8%)	719 (11.2%)	
25–42 week	41 (0.6%)	6 (0.1%)	35 (0.5%)^*^	

*p < 0.05 for the comparison of the specific subgroup proportion between the GDM and non-GDM groups, based on post hoc tests with Bonferroni adjustment.

Continuous variables with normal distribution are presented as mean ± standard deviation; those with non-normal distribution are presented as median (interquartile range) or median (min, max). Categorical variables are presented as a number (percentage).

### 3.2 Association of folic acid supplementation with the GDM risk

**Figure 1:**
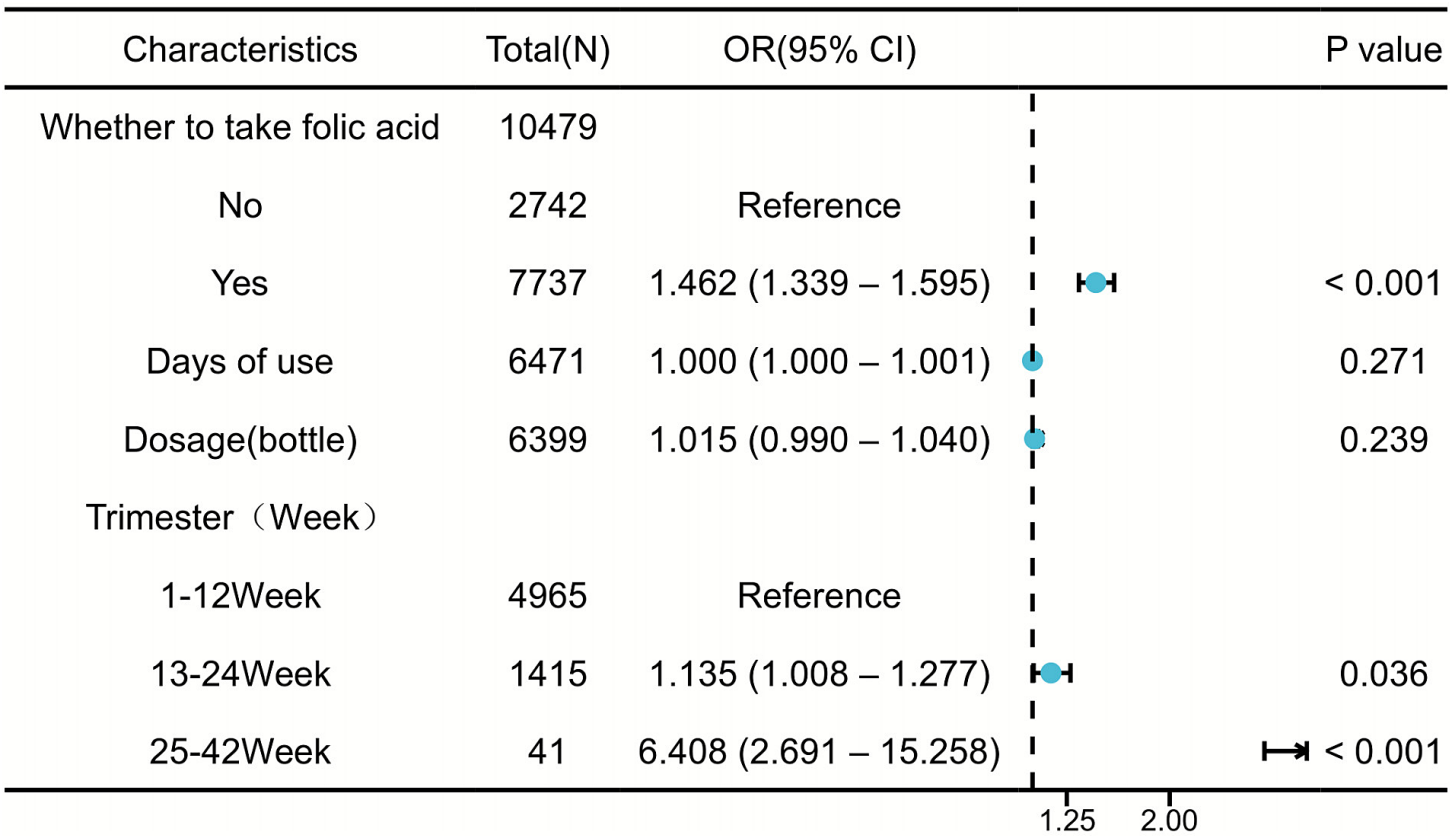
Forest plot of folate use and non-use in a retrospective cohort.

Folic acid supplement use (including compound preparations) was significantly associated with an increased GDM risk (adjusted OR = 1.462; 95% CI: 1.339–1.595; *p* < 0.001). After adjustment for demographic and anthropometric factors (Model 1: adjusted for age, height, and weight), the association remained significant (OR = 1.438; 95% CI: 1.312–1.576; *p* < 0.001). Model 2, which was additionally adjusted for education level and the timing of folic acid supplementation, yielded a similar result (OR = 1.421, 95% CI: 1.295–1.561; *p* < 0.001) (Table 2). These consistent findings indicate that the observed association is robust and unlikely to be explained by these measured confounders. Although neither the duration of use (*p* = 0.28) nor dosage (*p* = 0.24) was significantly associated with the GDM risk, both parameters exhibited consistent directional trends (duration: OR = 1.000, 95% CI: 1.000–1.001; dosage: OR = 1.015, 95% CI: 0.990–1.040) (Figure 1). Subgroup analyses based on the gestational period of supplement initiation revealed significantly increased GDM risks in both the mid-pregnancy (13–24 weeks; adjusted OR = 1.135; 95% CI: 1.008–1.277; *p* = 0.036) and late-pregnancy (25–42 weeks; adjusted OR = 6.408; 95% CI: 2.691–15.258; *p* < 0.001) groups compared with the early pregnancy initiation group (1–12 weeks). Notably, the sample size of the late-pregnancy subgroup was smaller, which may have limited the statistical power and amplified the effect estimates.

**Table 2:** Association between folic acid supplementation and gestational diabetes mellitus risk in the hospital-based retrospective cohort study.

Model description	OR (95% CI)	*p* value
Crude model	1.462 (1.339–1.595)	<0.001
Model 1	1.438 (1.312–1.576)	<0.001
Model 2	1.421 (1.295–1.561)	<0.001

Crude model adjusts for: none.

Model 1 adjusts for age, height, and weight.

Model 2 adjusts for Model 1 and education level and trimester.

### 3.3 Genetic evidence for an association between folic acid supplementation and the GDM risk

**Figure 2:**
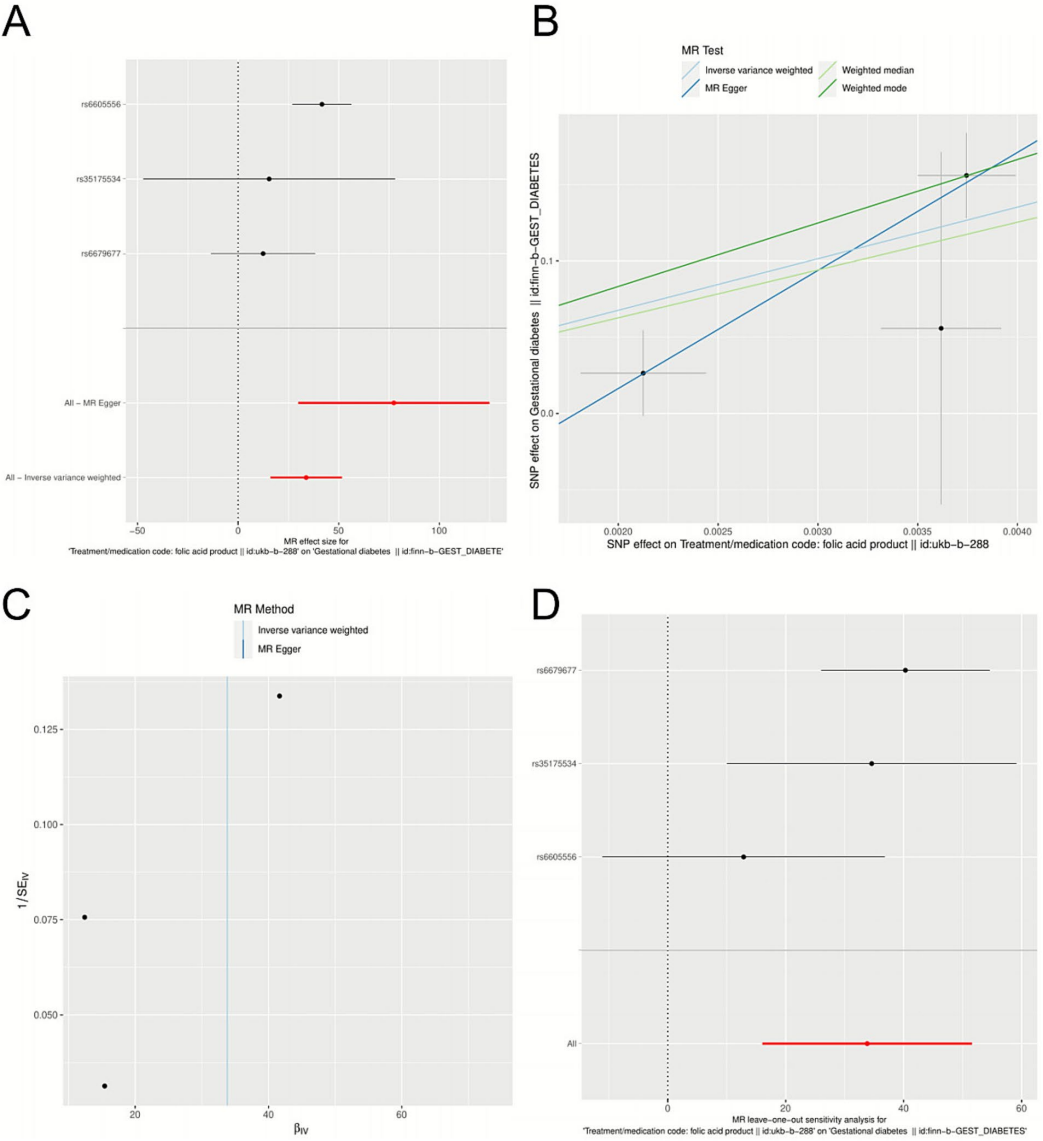
The result of Mendelian randomization. **(A)** Forest map of folic acid exposure and gestational diabetes mellitus. **(B)** Correlation scatter plots of different algorithms. **(C)** Heterogeneity analysis. **(D)** One method sensitivity analysis.

Mendelian randomization analysis using genetic variants associated with folic acid supplementation (all F statistics > 10) indicated a positive association between genetically predicted folate intake and the GDM risk. A detailed summary of each IV, including β, SE, R^2^, F-statistics, and pleiotropy queries, is provided in Supplementary Table S2 to improve transparency and reproducibility. The inverse variance weighted method showed a significant effect (β = 33.81, SE = 9.074, *p* = 0.00019), with the other MR methods displaying directionally consistent estimates (Figure 2A; Supplementary Table S1). Sensitivity analyses supported the robustness of these findings, showing no substantial heterogeneity (MR–Egger: Q = 0.5786, *p* = 0.4469) and (IVW: Q = 4.049, *p* = 0.132) or significant horizontal pleiotropy (MR–Egger intercept *p* = 0.314) (Figures 2B–C; Supplementary Table S3). We rescaled the MR effect estimate using behaviorally meaningful increments to improve interpretability. When expressed per 1% increase in the odds of folic acid supplementation, the IVW method yielded an OR of 1.40 (95% CI 1.17–1.67, *p* < 0.001). The leave-one-out analysis confirmed that no single genetic variant drove the association (all *p* < 0.05) (Figure 2D). A full summary of pleiotropy and heterogeneity assessments, including MR-PRESSO and PhenoScanner screening, is provided in Supplementary Table S4. The observed association provides genetic support for a potential causal link between folic acid supplement use behavior and GDM, which is consistent with a causal interpretation. However, the MR exposure reflects genetically predicted folic acid product behavior rather than folic acid bioavailability.

### 3.4 Characteristics of the study population in the NHANES

**Figure 3:**
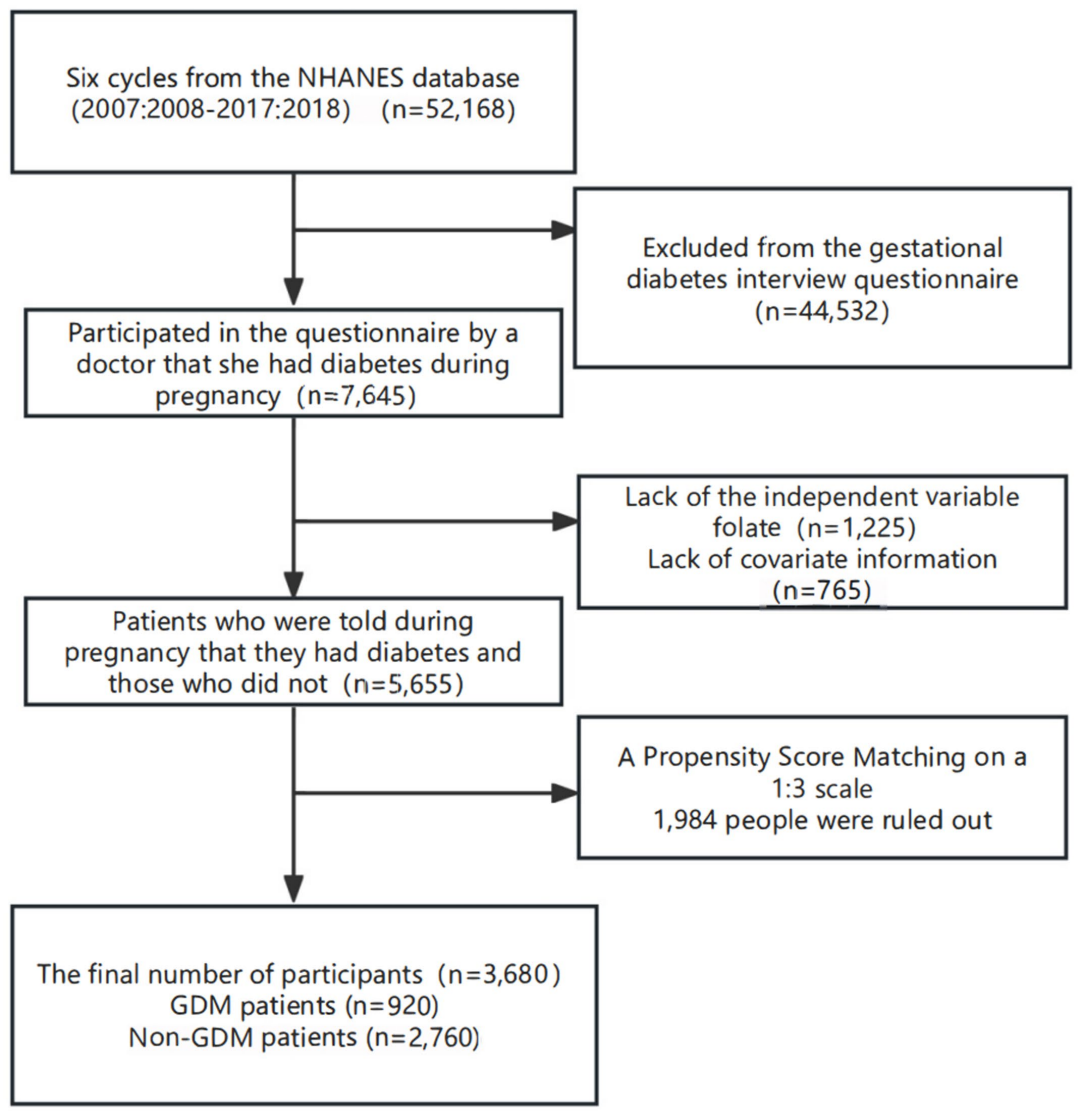
Inclusion flow chart of the US National Nutrition and Survey study population.

From 52,168 initially enrolled participants, we included 3,680 individuals (920 GDM patients and 2,760 controls) after exclusion and 1:3 propensity score matching (Figure 3). Baseline characteristics revealed significant differences between the GDM and non-GDM groups in terms of glycemic control (HbA1c levels), folate intake measures (total folate intake, dietary folate intake, and DFEs), race or ethnic origin distribution, dyslipidemia, and selenium levels (all *p* < 0.05) (Table 3). Specifically, the GDM group presented elevated HbA1c levels and consistently increased folate intake across all the groups. No significant differences were observed in total energy intake, vitamin B12 status, or the levels of other trace elements (iron or zinc). These findings indicate that the GDM population is characterized by both metabolic disturbances and increased folate exposure.

**Table 3:** Univariate analysis of patients with and without gestational diabetes mellitus in the NHANES cohort.

Character	Total (*N* = 3,680)	Without GDM (*N* = 2,760)	GDM (*N* = 920)	*P*-value
Age	29.00 (24.00, 33.00)	29.00 (24.00, 33.00)	28.00 (23.00, 33.00)	0.250
Weight	77.60 (64.90, 94.20)	77.10 (64.60, 93.80)	79.00 (66.20, 96.00)	0.180
Height	161.50 (156.70, 165.80)	161.60 (156.60, 165.80)	161.40 (157.10, 165.90)	0.610
BMI	29.92 (25.09, 35.90)	29.80 (24.90, 35.80)	30.40 (25.50, 36.30)	0.300
HbA1c	5.50 (5.20, 5.70)	5.40 (5.20, 5.70)	5.60 (5.30, 6.10)	<0.001
Total folate (mcg)	312.00 (215.00, 443.00)	303.00 (209.00, 436.00)	333.00 (234.00, 457.00)	0.020
Food folate (mcg)	168.00 (113.00, 241.00)	166.00 (111.00, 235.00)	174.00 (117.00, 248.00)	0.041
Folate as dietary folate equivalents (mcg)	401.00 (266.00, 579.00)	391.00 (259.00, 567.00)	426.00 (291.00, 597.00)	0.020
Vitamin B12 (mcg)	3.17 (1.76, 5.19)	3.26 (1.82, 5.29)	2.83 (1.63, 4.74)	0.010
Added vitamin B12 (mcg)	0 (0, 0.1)	0 (0, 0)	0.01 (0, 0.1)	0.002
Energy (Kcal)	1771.00 (1340.00, 2268.00)	1764.00 (1337.00, 2266.00)	1793.00 (1346.00, 2270.00)	0.320
Iron (mg)	11.49 (8.10, 16.04)	11.43 (8.03, 15.98)	11.95 (8.51, 16.18)	0.060
Zinc (mg)	8.73 (6.13, 12.10)	8.66 (6.02, 12.17)	9.09 (6.62, 11.80)	0.072
Selenium (mg)	89.80 (64.30, 121.20)	88.20 (63.10, 119.80)	96.20 (68.00, 126.70)	0.013
BMI classification (kg/m^2^)				<0.001
Underweight (<18.5 kg/m^2^)	40 (8196.75)	32 (1.57)	8 (1.01)	
Normal (18.5–24.9 kg/m^2^)	594 (103498.16)	451 (17.96)	143 (18.18)	
Overweight (25.0–29.9 kg/m^2^)	713 (109702.66)	550 (19.82)	163 (16.95)^*^	
Obese Class I (30.0–34.9 kg/m^2^)	556 (86460.08)	350 (12.70)	206 (22.05)^*^	
Obese Class II (35.0–39.9 kg/m^2^)	206 (31978.39)	150 (5.44)	56 (5.95)	
Obese Class III (≥40.0 kg/m^2^)	1,571 (234560.50)	1,171 (40.51)	400 (41.81)^*^	
Alcohol consumption				0.830
Former	490 (70569.66)	369 (12.71)	121 (12.45)	
Heavy	746 (116392.49)	567 (21.49)	179 (18.95)	
Mild	954 (161051.39)	706 (27.98)	248 (31.43)	
Moderate	745 (132495.82)	569 (24.25)	176 (22.21)	
Never	652 (77713.09)	479 (13.57)	173 (14.95)	
Smoking status				0.941
Former	576 (102645.53)	428 (17.24)	148 (19.76)	
Never	2,317 (347924.59)	1,741 (60.86)	576 (59.81)	
Now	785 (123646.50)	589 (21.91)	196 (20.43)	
Education				0.492
Below junior high school graduation	899 (98599.05)	686 (17.13)	213 (17.26)	
College or above	2,009 (350957.51)	1,487 (60.64)	522 (62.46)	
High school graduation	772 (124839.98)	587 (22.23)	185 (20.27)	
Race or ethnic origin				0.425
Non-Hispanic Black	841 (79758.87)	673 (14.95)	168 (10.75)	
Non-Hispanic White	1,246 (341560.39)	910 (58.85)	336 (61.28)	
Other Race/Ethnicity	1,593 (153077.29)	1,177 (26.20)	416 (27.97)	
Hypertension				0.090
No	2,450 (394024.81)	1,868 (69.17)	582 (66.91)	
Yes	1,230 (180371.74)	892 (30.83)	338 (33.09)	
Hyperlipidemia				0.002
No	1,110 (175574.21)	887 (31.99)	223 (26.37)^*^	
Yes	2,570 (398822.33)	1,873 (68.01)	697 (73.63)^*^	

*p < 0.05 for the comparison of the specific subgroup proportion between the GDM and non-GDM groups, based on post hoc tests with Bonferroni adjustment.

Continuous variables with normal distribution are presented as mean ± standard deviation; those with non-normal distribution are presented as median (interquartile range) or median (min, max). Categorical variables are presented as a number (percentage).

### 3.5 A potential link between folate intake and the GDM risk

Quartile analyses revealed a significant dose-dependent relationship between folate intake and the GDM risk. With respect to total folate intake, compared with participants in the lowest quartile (Q1), participants in Q3 (OR = 1.41, 95% CI: 1.08–1.84) and Q4 (OR = 1.42, 95% CI: 1.05–1.92) displayed a significantly elevated GDM risk. Similarly, DFE intake was associated with the highest risk in Q3 (OR = 1.61, 95% CI: 1.23–2.10), with a sustained increase observed in Q4 (OR = 1.42, 95% CI: 1.05–1.95). These associations remained statistically significant after comprehensive adjustment for demographic, metabolic, and nutritional covariates across all the models (all *p* < 0.05; Tables 4, 5), indicating a robust dose–response relationship between folate exposure and the GDM risk.

**Table 4:** Multivariate adjusted regression model of total folate intake and gestational diabetes mellitus in the NHANES cohort.

Character	Crude model		Model 1		Model 2		Model 3		Model 4	
Total folate	OR(95%CI)	*P*	OR(95%CI)	*P*	OR(95%CI)	*P*	OR(95%CI)	*P*	OR(95%CI)	*P*
Q1 (0, 206)	ref		ref		ref		ref		ref	
Q2 (206, 307)	1.08 (0.81, 1.44)	0.591	1.09 (0.81, 1.46)	0.583	1.08 (0.81, 1.44)	0.603	1.05 (0.79, 1.40)	0.738	0.98 (0.73, 1.32)	0.902
Q3 (307, 435)	1.41 (1.08, 1.84)	0.010	1.43 (1.07, 1.92)	0.022	1.45 (1.09, 1.93)	0.012	1.40 (1.05, 1.87)	0.022	1.39 (1.03, 1.88)	0.033
Q4 (435, 2,357)	1.42 (1.05, 1.92)	0.018	1.44 (1.04, 2.01)	0.031	1.47 (1.06, 2.03)	0.021	1.43 (1.04, 1.97)	0.032	1.44 (1.09, 1.90)	0.010

Crude model adjusts for none.

Model 1 adjusts for alcohol consumption, smoking status, glycohemoglobin.

Model 2 adjusts for alcohol consumption, smoking status, glycohemoglobin, race or ethnic origin, education.

Model 3 adjusts for alcohol consumption, smoking status, glycohemoglobin, race or ethnic origin, education, BMI classification, hyperlipidemia, hypertension, vitamin B12.

Model 4 adjusts for alcohol consumption, smoking status, glycohemoglobin, race or ethnic origin, education, BMI classification, hyperlipidemia, hypertension, vitamin B12, additional vitamin B12 supplementation, energy, iron, zinc, and selenium.

**Table 5:** Multifactor adjusted regression model of folate as dietary folate equivalent (DFE) and gestational diabetes mellitus in the NHANES cohort.

Character	Crude model		Model 1		Model 2		Model 3		Model 4	
DFE	OR(95%CI)	*P*	OR(95%CI)	*P*	OR(95%CI)	*P*	OR(95%CI)	*P*	OR(95%CI)	*P*
Q1 (0, 257)	ref		ref		ref		ref		ref	
Q2 (257, 397)	1.17 (0.85, 1.62)	0.330	1.13 (0.81, 1.57)	0.483	1.08 (0.78, 1.50)	0.640	1.09 (0.79, 1.51)	0.597	1.05 (0.75, 1.46)	0.781
Q3 (397, 563)	1.61 (1.23, 2.10)	<0.001	1.67 (1.26, 2.22)	<0.001	1.62 (1.22, 2.16)	<0.001	1.63 (1.22, 2.17)	<0.001	1.64 (1.22, 2.21)	<0.001
Q4 (563, 3,411)	1.42 (1.04, 1.95)	0.031	1.41 (1.01, 1.96)	0.038	1.37 (1.01, 1.86)	0.039	1.38 (1.01, 1.88)	0.072	1.39 (1.03, 1.88)	0.032

DFE, Folate as dietary folate equivalents.

Crude model adjusts for none.

Model 1 adjusts for alcohol consumption, smoking status, glycohemoglobin.

Model 2 adjusts for alcohol consumption, smoking status, glycohemoglobin, race or ethnic origin, education.

Model 3 adjusts for alcohol consumption, smoking status, glycohemoglobin, race or ethnic origin, education, BMI classification, hyperlipidemia, hypertension, vitamin B12.

Model 4 adjusts for Alcohol consumption, smoking status, glycohemoglobin, race or ethnic origin, education, BMI classification, hyperlipidemia, hypertension, vitamin B12, additional vitamin B12 supplementation, energy, iron, zinc, and selenium.

Stratified analyses showed that the association between folate intake and the GDM risk may be modified by metabolic comorbidities. The positive association was more pronounced in participants without hypertension, with a significantly increased risk in Q4 of total folate intake (OR = 1.48, 95% CI: 1.35–1.66; *p* < 0.001), while no significant association was observed in hypertensive individuals. Similarly, a significant interaction was observed with the hyperlipidemia status (P for interaction = 0.026), with stronger associations between DFE intake and the GDM risk detected in participants without hyperlipidemia (Q4 OR = 1.42, 95% CI: 1.04–1.93) (Table 6). These findings indicate that the folate–GDM relationship may be modified by the metabolic status, with the association appearing more apparent in individuals without these conditions.

**Table 6:** Subgroup analysis of the linear regression model relationship between total folate intake and gestational diabetes mellitus in the NHANES cohort.

Character	Q1	Q2	*p*	Q3	*p*	Q4	*p*	p for trend (character2integer)	p for interaction
		OR(95%CI)		OR(95%CI)		OR(95%CI)			
Smoking status									0.717
Never	ref	1.028 (0.705, 1.499)	0.886	1.453 (0.998, 2.114)	0.051	1.444 (0.993, 2.100)	0.054	0.021	
Now	ref	1.092 (0.677, 1.760)	0.716	1.051 (0.623, 1.773)	0.850	1.017 (0.581, 1.782)	0.951	0.944	
Former	ref	1.322 (0.661, 2.643)	0.425	1.896 (0.926, 3.885)	0.080	1.932 (0.885, 4.216)	0.097	0.067	
Alcohol consumption									0.209
Mild	ref	1.150 (0.648, 2.040)	0.630	1.736 (1.037, 2.904)	0.036^*^	1.330 (0.733, 2.415)	0.344	0.229	
Moderate	ref	2.106 (1.118, 3.968)	0.022^*^	1.725 (0.930, 3.199)	0.083	1.565 (0.824, 2.971)	0.168	0.361	
Heavy	ref	0.601 (0.314, 1.148)	0.121	1.064 (0.600, 1.888)	0.830	1.308 (0.683, 2.503)	0.414	0.197	
Former	ref	0.585 (0.270, 1.265)	0.169	1.010 (0.503, 2.031)	0.977	1.162 (0.507, 2.661)	0.718	0.530	
Never	ref	1.071 (0.553, 2.073)	0.837	1.309 (0.653, 2.621)	0.443	1.867 (0.975, 3.574)	0.059	0.055	
BMI classification (kg/m^2^)									0.993
Underweight (<18.5 kg/m^2^)	ref	0.920 (0.014, 65.480)	0.952	0.765 (0.007, 102.280)	0.870	1.390 (0.044, 45.320)	0.782	0.831	
Normal (18.5–24.9 kg/m^2^)	ref	1.410 (0.738, 2.694)	0.293	1.376 (0.668, 2.836)	0.382	1.474 (0.780, 2.784)	0.228	0.337	
Overweight (25.0–29.9 kg/m^2^)	ref	0.920 (0.454, 1.864)	0.810	1.790 (0.892, 3.591)	0.098	1.438 (0.741, 2.788)	0.279	0.107	
Obese Class I (30.0–34.9 kg/m^2^)	ref	1.101 (0.538, 2.256)	0.791	1.430 (0.750, 2.727)	0.274	1.632 (0.795, 3.351)	0.183	0.095	
Obese Class II (35.0–39.9 kg/m^2^)	ref	0.789 (0.320, 1.945)	0.604	0.936 (0.427, 2.050)	0.868	1.285 (0.521, 3.168)	0.589	0.324	
Obese Class III (≥40.0 kg/m^2^)	ref	1.205 (0.752, 1.931)	0.436	1.588 (1.024, 2.462)	0.039^*^	1.812 (1.152, 2.852)	0.010^*^	0.003	
Education									0.849
Below junior high school graduation	ref	0.806 (0.473, 1.374)	0.424	1.034 (0.600, 1.784)	0.902	1.288 (0.754, 2.200)	0.350	0.289	
College or above	ref	1.120 (0.752, 1.667)	0.574	1.425 (0.965, 2.104)	0.075	1.451 (0.996, 2.112)	0.052	0.026	
High school graduation	ref	1.226 (0.697, 2.158)	0.475	1.774 (1.026, 3.068)	0.041^*^	1.420 (0.773, 2.611)	0.255	0.130	
Race or ethnic origin									0.810
Non-Hispanic Black	ref	1.050 (0.680, 1.620)	0.820	1.450 (0.960, 2.190)	0.080	1.520 (0.980, 2.360)	0.060	0.030	
Non-Hispanic White	ref	1.180 (0.730, 1.910)	0.500	1.320 (0.840, 2.080)	0.230	1.020 (0.640, 1.630)	0.940	0.880	
Other Race/Ethnicity	ref	0.950 (0.430, 2.100)	0.900	1.050 (0.470, 2.340)	0.900	1.350 (0.600, 3.050)	0.470	0.400	
Hypertension									0.208
No	ref	0.969 (0.683, 1.377)	0.861	1.442 (1.030, 2.019)	0.033^*^	1.573 (1.098, 2.254)	0.014^*^	0.003	
Yes	ref	1.308 (0.851, 2.011)	0.218	1.365 (0.871, 2.139)	0.172	1.129 (0.706, 1.806)	0.610	0.613	
Hyperlipidemia									0.028
No	ref	0.847 (0.497, 1.444)	0.537	1.437 (0.850, 2.429)	0.173	2.163 (1.261, 3.710)	0.006^*^	0.002	
Yes	ref	1.177 (0.864, 1.604)	0.298	1.417 (1.043, 1.926)	0.026^*^	1.201 (0.841, 1.714)	0.311	0.211	

*p < 0.05 for the comparison of the specific quartile (Q2, Q3, or Q4) with the reference quartile (Q1) within each stratified subgroup.

### 3.6 Non-linear models suggest a threshold effect of folate intake on the GDM risk

**Figure 4:**
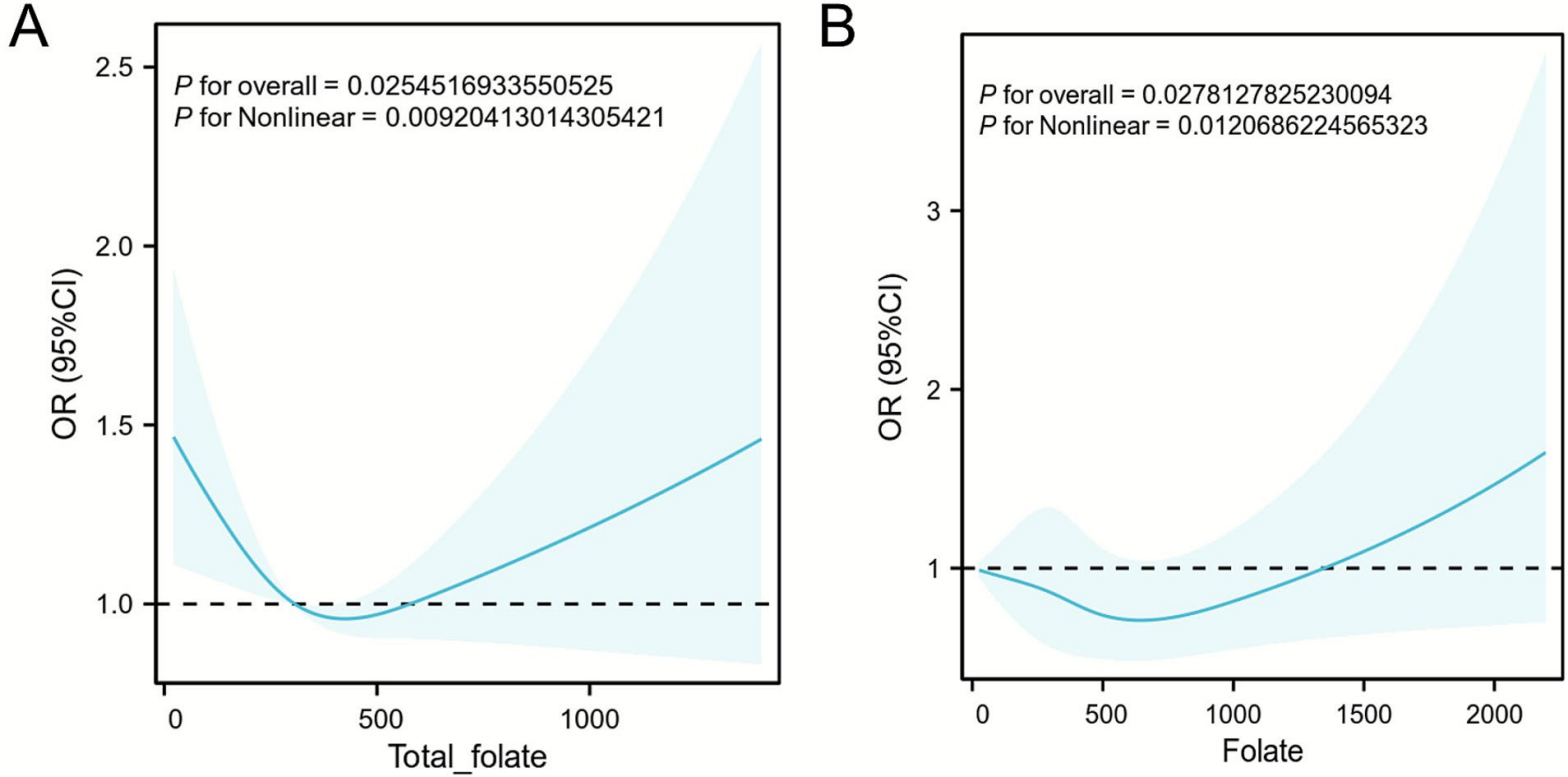
Different forms of folate and gestational diabetes mellitus. **(A)** Restricted cubic splines of total folate intake and gestational diabetes mellitus. **(B)** restricted cubic splines of dietary folate equivalent intake and gestational diabetes mellitus.

The restricted cubic spline analysis revealed a significant U-shaped relationship between total folate intake and the GDM risk (P for non-linearity < 0.05). The GDM risk initially decreased with increasing folate intake, reaching its lowest point at approximately 445 mcg/day, beyond which the risk progressively increased. This pattern suggests a critical threshold of approximately 445 mcg/day for total folate intake (Figure 4A). A similar non-linear pattern was observed for dietary folate equivalents (DFEs). Compared with the lowest quartile (Q1), both Q3 (397–563 mcg/d; OR = 1.61; 95% CI: 1.23–2.10) and Q4 (>564 mcg/d; OR = 1.42; 95% CI: 1.04–1.95) showed a significantly increased GDM risk, with the highest risk observed in Q3 (Figure 4B). This threshold should be interpreted cautiously, as it is derived from cross-sectional data. The RCS models demonstrated satisfactory goodness-of-fit, with likelihood ratio tests confirming the superior fit of non-linear specifications over linear models (total folate: χ^2^ = 12.26, *p* = 0.002; DFE: χ^2^ = 15.82, *p* = 0.0004). Sensitivity analyses using different knot configurations (3–5 knots placed at standard quantiles) yielded consistent results, supporting the robustness of the identified non-linear associations. Complete details of the statistical analysis are provided in Supplementary Table S5.

## 4 Discussion

### 4.1 Interpretation of the findings across diverse populations

This study integrates data from a Chinese cohort, European genetic databases, and the U. S. NHANES survey. While this multipopulation design strengthens the study by providing consistent evidence from different settings, it also introduces population heterogeneity (32, 33). We acknowledge that the genetic background, dietary patterns, and folic acid fortification policies differ across these populations, which may influence the relationship between folate and GDM. For example, the Mendelian randomization result is based on European genetic data, whereas the NHANES analysis reflects the context of mandatory folic acid fortification in the U. S. In contrast, China lacks such a policy (34). These differences suggest that the observed associations may not be directly generalizable across populations. Therefore, the main goal of this study is not to establish a universal effect but to report a consistent association across methodologically diverse analyses and distinct populations. This consistency suggests a potential biological link between folate and GDM. Future studies should focus on population-specific designs and explore gene–environment interactions to better understand these relationships in different settings. We recognize that only three SNPs were available as genome-wide significant predictors of folic acid supplementation behavior. This result is not unexpected given the behavioral nature of the exposure, which typically has low heritability. Importantly, MR guidelines emphasize that the number of SNPs is less critical than their individual strength and validity. All three SNPs had F-statistics >10 and passed pleiotropy screening, supporting their relevance as instruments. Although fewer IVs reduce the opportunity for overidentification tests, they also lower the risk of including invalid instruments. Our findings, therefore, rely on a focused set of robust genetic predictors rather than a broader set that may introduce additional bias. Our Mendelian randomization analysis provides complementary, independent evidence. Genetic instruments predict folic acid supplementation behavior rather than circulating folate levels and are unaffected by potential confounding from the supplement type. The consistent positive association observed in the MR analysis further supports a potential causal link between folic acid supplementation and the GDM risk, reinforcing the robustness of our findings.

### 4.2 Consistent association of folic acid and folate intake with the GDM risk across populations

This study initially suggested a potential relationship between folic acid supplement use and the risk of GDM based on data from a retrospective cohort from Jiangsu Province, China. The observed elevated risk with late-pregnancy folic acid supplementation likely reflects reverse causality or confounding by indication rather than a direct effect of folic acid use initiated at this stage. Women who begin taking folic acid supplements in the second or third trimester may have pre-existing risk factors or complications that prompt later supplementation. We acknowledge that the exposure variable “folic acid use” was defined in a binary manner, which limited our ability to differentiate between single folic acid and multivitamin preparations or to assess dose–response relationships. However, because most participants reported using single folic acid tablets, this limitation likely led to a conservative rather than exaggerated estimate of the true association. An MR analysis was conducted using the genetic variants associated with folic acid products as instrumental variables to further explore the potential link between genetically predicted folic acid levels and GDM. The MR results indicated that higher genetically predicted folic acid product levels were associated with an increased risk of GDM (beta = 33.81, se = 9.074, *p* < 0.001), which may lend some support to a potential causal role of folic acid supplementation in the GDM risk. We rescaled the MR effect estimate using behaviorally meaningful increments to improve interpretability. When expressed per 1% increase in the odds of folic acid supplementation, the IVW method yielded an OR of 1.40 (95% CI 1.17–1.67, *p* < 0.001). Although the MR analysis helps mitigate confounding, the results should still be interpreted with caution. The discrepancy in effect size between the MR and observational findings should not be interpreted as an inconsistency. Fundamentally, MR estimates represent the lifetime effect of a genetically influenced propensity toward folic acid supplementation behavior, whereas estimates from the retrospective cohort and NHANES reflect short-term behavioral supplementation during pregnancy. These two sources of evidence operate on different exposure scales (lifelong liability vs. pregnancy-limited intake); therefore, their effect sizes are not expected to match numerically. Moreover, observational analyses reflect modifiable health behaviors. These biological and methodological differences help explain why MR may produce larger liability estimates. Although folic acid supplementation behavior is partly influenced by environmental and socioeconomic factors, previous studies have shown that supplement use behaviors exhibit measurable heritability through genetic determinants related to health-seeking behaviors, nutrient metabolism, and micronutrient transport pathways. The three SNPs selected from UKB-B-288 represent the strongest genome-wide signals associated with folic acid supplement use. Their inclusion aligns with MR methodological recommendations for behavioral exposures, where a small number of robust SNPs with F-statistics >10 can be considered adequate for a causal inference.

Further validation was performed using NHANES data from the United States, which include detailed information on folate intake. The NHANES results also indicated a non-linear relationship between folate intake and the GDM risk. When folate intake was grouped by quartiles, a non-linear relationship was observed. Subgroup analyses indicated that the non-linear relationship was consistent across populations with hypertension and hyperlipidemia. These findings remained robust after adjusting for various potential confounders, such as alcohol consumption, smoking status, HbA1c level, race or ethnic origin, education level, BMI categories, hyperlipidemia, hypertension, and vitamin B12 level. The analysis of the timing of folic acid supplementation revealed potentially higher GDM risks when initiation occurred in mid- or late pregnancy than in early pregnancy. These findings suggest that the timing of supplementation may be a critical factor. The introduction of folic acid later in pregnancy, when the maternal metabolic load increases, has been proposed to influence insulin sensitivity (35, 36). However, the notably high risk observed in the late-pregnancy group requires a cautious interpretation due to the limited sample size and warrants further investigation. Additionally, the consistent application of precise terminology throughout this study—distinguishing “folic acid” (supplemental) from “folate” (dietary)—has strengthened our ability to draw meaningful conclusions about their potentially distinct relationships with the GDM risk. This terminological rigor enhances the interpretability of our findings from different methodological approaches and data sources. Furthermore, our simultaneous examination of three distinct folate metrics—total folate intake, dietary folate intake, and DFEs—provides complementary insights but also necessitates a careful interpretation. The differential associations observed across these metrics likely reflect both true biological differences in the effects of various folate forms and the methodological particularities of each measure, particularly the bioavailability adjustment inherent in DFE calculations.

### 4.3 Non-linear association and dual effect of folate intake on the GDM risk

As an essential B vitamin, folate plays a crucial role in DNA methylation, amino acid metabolism, and oxidative stress regulation. Recently, the relationship between folate supplementation and the GDM risk has attracted increasing attention. However, existing studies have yielded conflicting results. Some studies suggest that moderate folate supplementation may reduce the risk of GDM by improving insulin sensitivity, reducing inflammation, and mitigating oxidative stress (37). In contrast, other studies have indicated that excessive folate intake may be associated with insulin resistance and glucose abnormalities, thereby increasing the risk of GDM (38). Our dose–response analysis revealed a non-linear, U-shaped relationship between folate intake and the GDM risk, with an inflection point observed at approximately 445 mcg/day, suggesting a potential threshold beyond which the benefit of higher intake may decrease. These findings are consistent with the weak recommendation in the “Chinese Diabetes Medical Nutrition Therapy Guidelines (2022 version)” (39), which suggests that daily folate supplementation of 400 mcg in early pregnancy may be beneficial, but exceeding 800 mcg could increase the risk. Although some studies have reported no significant association between folic acid levels and GDM, our findings align with those of several studies reporting positive associations. For instance, Li et al. performed a prospective cohort study and reported that sustained high-dose folic acid use (≥800 mcg/day) was associated with a twofold increase in the GDM risk (adjusted OR = 2.09) (9). This collective evidence suggests that the association between folic acid and GDM can be substantial under specific exposure conditions, providing a plausible context for our observed effect size. Similarly, several studies have shown a non-linear association between folate intake and other diseases, such as osteoporosis, dyslipidemia, and poor cognitive function. In osteoporosis research, folate deficiency exacerbates bone loss by impairing homocysteine (Hcy)-mediated collagen crosslinking, whereas excessive intake of folate competitively inhibits zinc-dependent alkaline phosphatase, impairing bone mineralization (40). Similarly, in terms of cognitive function, a “U-shaped” relationship between folate levels and neuroprotective effects has been observed, suggesting a metabolic saturation threshold for epigenetic regulation (41). This phenomenon reveals a classic double-edged sword effect of folate intake. Under specific physiological conditions, appropriate folate intake can positively regulate health; however, when intake deviates from the normal range, metabolic disruptions may occur that interfere with normal physiological processes. The findings of our study are consistent with the results of these studies and further support the dose-dependent relationship between folate levels and GDM, highlighting the importance of monitoring appropriate folate intake. The lack of a clear dose–response relationship in the retrospective cohort is likely due to the limited precision of the exposure measurement, whereas NHANES data provide high-resolution continuous estimates of total folate intake, dietary folate intake, and dietary folate equivalents (DFEs), enabling the detection of non-linear patterns (~445 mcg/day). The threshold of ~445 mcg/day for total folate intake from the NHANES analysis is hypothesis-generating and based on cross-sectional data. Clinical decisions should not be made without confirmation from prospective studies. Any potential GDM risk must be weighed against the proven benefits of folic acid for preventing neural tube defects, highlighting the need for further research on optimal folate intake during pregnancy.

### 4.4 Potential mechanisms linking folate to GDM

The potential mechanisms linking folate to GDM are multifaceted and involve one-carbon metabolism, inflammatory pathways, and interactions with vitamin B12, although direct evidence from our study is limited. First, folate plays a central role in one-carbon metabolism, regulating the expression of insulin-related genes through DNA methylation and thus affecting insulin sensitivity (42). Second, abnormal folate levels may affect homocysteine (Hcy) metabolism, leading to elevated Hcy levels, which have been linked to increased insulin resistance and diabetes risk (43–45). Additionally, folate may improve insulin resistance by enhancing antioxidant and anti-inflammatory pathways, thus improving pancreatic β-cell function. However, excessive folate may disrupt the methylation balance, leading to abnormal gene expression and affecting glucose metabolic homeostasis (46). From a mechanistic perspective, folate may have a dual effect on glucose metabolism. Adequate folate intake supports normal homocysteine metabolism, whereas excessive supplementation may disrupt one-carbon metabolism and exacerbate vitamin B12 deficiency-related insulin resistance. Animal studies have shown that folic acid can activate the AMP-activated protein kinase (AMPK) pathway, a key regulator of glucose and lipid metabolism; however, high folate levels may amplify metabolic stress when vitamin B12 levels are insufficient. Collectively, these findings suggest a potential U-shaped or dose-dependent relationship between folic acid exposure and the GDM risk, emphasizing the need for a cautious interpretation based on the dosage and population characteristics.

The divergent associations observed for supplemental and dietary folate intake invite a mechanistic exploration beyond the traditional focus on one-carbon metabolism. These pathways may intersect with oxidative stress and endothelial dysfunction, which are established pillars of the pathogenesis of metabolic diseases (47). For instance, high-dose folic acid supplementation could lead to the accumulation of unmetabolized folic acid (UMFA), a compound implicated in promoting oxidative stress and impairing endothelial function. This perspective is supported by a growing body of literature that recasts folate metabolism within a broader framework of vascular health, highlighting its interplay with one-carbon and homocysteine-related mechanisms that directly influence insulin signaling and vascular integrity (6, 43). This expanded model offers a more holistic biological rationale for our results.

In addition, this study revealed that folate levels were significantly increased in the GDM group, whereas vitamin B12 levels were significantly reduced (total folate intake: 333.00 vs. 303.00 mcg, *p* = 0.02; vitamin B12: 2.83 vs. 3.26 mcg, *p* = 0.01) (Table 3). While this association is notable, it is an observational finding from our baseline data and does not support definitive causal or mechanistic conclusions. According to some studies, excessive folate intake leads to the accumulation of unmetabolized folate (UMFA), which interferes with vitamin B12-dependent methionine synthase (MTR) function, resulting in elevated serum vitamin B12 levels but decreased tissue utilization, thereby exacerbating glucose metabolic disturbances through the activation of the NF-κB inflammatory pathway and mitochondrial oxidative damage (48–50). Our results may provide some preliminary support for the accumulating evidence on this topic. Notably, these mechanistic pathways remain speculative in the context of our study. Due to the lack of functional biomarkers such as serum folate, UMFA and homocysteine levels, direct verification is not possible. Therefore, future studies should incorporate serial measurements of these functional biomarkers to properly assess the temporal dynamics and biological consequences of this nutrient imbalance.

### 4.5 Limitations, strengths, and future research directions

Our study has several limitations that should inform the interpretation of its findings. First, the binary characterization of folic acid supplementation in our primary cohort, while pragmatic, obscures critical details on the dosage, duration, and formulation. This lack of granularity fundamentally limits our ability to explore dose–response relationships and may mask important nuances in exposure. Second, the use of the self-reported GDM status in the NHANES analysis, although a common necessity in large national surveys, introduces a tangible risk of recall bias and outcome misclassification. Additionally, some subgroup analyses were restricted to participants with complete data on all covariates. This complete-case approach led to reductions in the effective sample size for specific analyses, which may limit the statistical power and generalizability of those findings. Third, while Mendelian randomization represents a powerful approach to infer causality, our analysis was constrained by the limited availability of genetic instruments. The use of only three SNPs not only reduces statistical power but also complicates a thorough assessment of pleiotropy, a key assumption of the method. Finally, the absence of direct biomarkers (e.g., serum folate, UMFA, and homocysteine levels) means that our discussion of the underlying mechanisms remains speculative and is grounded in plausibility rather than direct physiological evidence. Future research incorporating these biomarkers is essential to move from an association to a mechanism. The strengths of this study include three main aspects. First, the multidesign approach combined data from Chinese, European, and U. S. populations, providing a large sample size and enabling external validation. Second, the restricted cubic spline analysis revealed a non-linear relationship between folate intake and the GDM risk and identified a potential threshold, providing insights beyond linear assumptions. Third, cross-validation across datasets increased the reliability of the findings. Future research should prioritize analyses of longitudinal cohorts with serial biomarker measurements and randomized controlled trials to definitively establish causality and explore the dynamic relationships among folate levels, vitamin B12 levels, and the GDM risk.

## 5 Conclusion

This multicomponent study indicates that both supplemental folic acid intake and dietary folate intake are associated with an increased risk of gestational diabetes mellitus (GDM), revealing a potential non-linear dose–response relationship. Analyses using complementary approaches, including clinical observational data, genetic instrumental variable methods, and nationally representative nutritional surveys, consistently indicate that both supplemental folic acid and dietary folate intake may be associated with a higher GDM risk. While this triangulation of evidence suggests a potential effect of supplemental versus dietary folate on the GDM risk, these findings must be interpreted with caution. The inherent limitations of observational data, the constrained number of genetic instruments, and the reliance on self-reported diagnoses collectively necessitate confirmation in future studies designed specifically to assess causal inference. These findings highlight the need for further investigations into the roles of folic acid and dietary folate intake in GDM development. Future rigorously designed prospective studies or randomized controlled trials should be conducted to confirm these observations, clarify potential causality, and establish optimal intake ranges across diverse populations using standardized folate assessment methodologies.

## Data Availability

Publicly available datasets were analyzed in this study. This data can be found here in the NHANES database (https://wwwn.cdc.gov/nchs/nhanes/) and GWAS Project (https://gwas.mrcieu.ac.uk/).
